# Murinization of an anti-GITR IgG2c antibody DTA-1 inhibits immunogenicity-mediated anaphylaxis in C57BL/6J mice

**DOI:** 10.1186/2051-1426-3-S2-P182

**Published:** 2015-11-04

**Authors:** Hamsell Alvarez, Nicole Belmar, Melvin Fox, Josue Samayoa, Sarah Chan, Diane Hollenbaugh, Fiona Harding

**Affiliations:** 1 AbbVie Biotherapeutics, Redwood City, CA, USA

## 

Recent advances in Immuno-Oncology (IO) have shown that the immune system can be activated to induce long term durable anti-tumor responses. For IO drug development, immune activation is typically explored using rat surrogate antibodies in immunocompetent mouse models. While these models can be used to show efficacy in vivo, the development of anti-drug immune responses to experimental protein-based therapeutics can arise. Immunogenicity of surrogate antibodies may therefore represent an important obstacle to the evaluation of the anti-tumor efficacy of immune-modulator antibodies in syngeneic models.

A recent publication has shown that the surrogate rat IgG2b/λ GITR agonistic antibody DTA-1 can induce the development of anaphylaxis in mice upon repeated intraperitoneal dosing (3rd i.p. ≥ 1.0 mg/kg). Total IgE and anti-rat variable/Fc domain anti-drug IgG1 and IgG2b antibodies (ADAs) were detected in serum. Low levels of DTA-1 specific anti-idiotypic ADAs were also detected. The development of anaphylaxis was suggested to be due to GITR as a target. An alternative hypothesis is the potential antigenicity of the therapeutic itself. To this end, chimerized (rat V domains/mouse constant regions) and murinized (95% mouse sequence) DTA-1-based surrogate antibodies with a murine IgG2c heavy chain isotype were created. Murinized IgG2c/λ DTA-1 (EC50 ~ 1.0 nM) and chimeric IgG2c/κ DTA-1 (EC50 ~ 1.3 nM) showed similar cell surface GITR-binding affinity as the parental rat antibody (EC50 ~ 0.96 nM). Chimerization and murinization of DTA-1 did not affect GITR-induced T cell proliferation (3H-thymdine incorporation), cytokine release (IFNγ, IL-2), and viability. Murinized IgG2c/λ DTA-1 (EC50 ~ 0.06 μg/ml) and chimeric IgG2c/κ DTA-1 (EC50 ~ 0.05 μg/ml) showed similar NFκB signaling in vitro as the parental rIgG2b/λ DTA-1 (EC50 ~ 0.07 μg/ml). Finally, treatment of C57BL/6J mice with the chimerized and murinized DTA-1 antibodies on a C57BL/6J-matched IgG2c isotype resulted in reduced development and severity of anaphylaxis (3 doses, i.p., 3.0 mg/kg) as measured by drop of body temperature, behavioral effects, serum IgE and ADA levels. These results suggest that careful murinization and the selection of a strain-matched heavy chain isotype constitute important factors for the generation of ideal surrogate antibodies for testing IO mechanisms in vivo.

## Disclosures

All authors are employees of AbbVie. The design, study conduct, and financial support for this research was provided by AbbVie. AbbVie participated in the interpretation of data, review, and approval of the publication.

